# Nicotinamide potentiates amphotericin B activity against *Candida albicans*

**DOI:** 10.1080/21505594.2022.2119656

**Published:** 2022-09-06

**Authors:** Yu Yan, ZeBin Liao, Juan Shen, ZhenYu Zhu, YingYing Cao

**Affiliations:** a Shanghai Skin Disease Hospital, School of Medicine, Tongji University, Shanghai, China; b Shanghai Engineering Research Center for Topical Chinese Medicine, Shanghai, China; c School of Pharmacy, Second Military Medical University, Shanghai, China; d Shanghai Pulmonary Hospital, School of Medicine, Tongji University, Shanghai, China

**Keywords:** Nicotinamide, amphotericin B, *C. albicans*, histone deacetylation, HST3

## Abstract

Amphotericin B (AmB) is a widely used antifungal agent especially for the therapy of systemic fungal infections. However, the severe side effects of AmB often leads to the premature termination of the treatment. So it is imperative to find the drugs that can both reduce the dosage and enhance the antifungal efficacy of AmB. Here we demonstrated that Nicotinamide (NAM), a cheap and safe vitamin, could enhance the antifungal activities of AmB. We demonstrated the synergistic interaction of NAM and AmB against *Candida albicans* as well as other *Candida* spp. and *Cryptococcus neoformans*. Moreover, NAM could enhance of the activity of AmB against biofilm. This enhancement was also observed in disseminated candidiasis *in vivo*. Our further study revealed that AmB could induce oxidative damage through the modification of histone acetylation. AmB could inhibit the expression of HST3, an H3K56 deacetylase in *C. albicans*. The immunoblotting test revealed excessive H3K56ac in AmB-treated fungal cells. Consistantly, the *hst3Δ* mutant displayed high sensitivity to AmB, while addition of NAM, an H3K56 deacetylation inhibitor, resulted in an even severe inhibition in the growth of this strain. These results indicated that AmB could execute antifungal activity via boosting H3K56ac which was mediated by HST3, and the mechanism for the synergistic interaction of NAM and AmB was based on exacerbating this process, which led to even excessive H3K56ac and oxidative damage. This finding provided theoretical basis for better understanding the antifungal mechanisms of AmB and clinical application of this drug.

## Introduction

Nowadays, with the development in the transplant surgery, the use of immunosuppresive agents and antibiotics, the frequency of fungal infections is rising. *Candida albicans* is a pathogenic fungi commonly isolated from patients with nosocomial infections. Invasive candidiasis is often accompanied with approximately 40% of mortality [1]. However, the treatment of fungal-associated diseases is challenged by the restricted number of antifungal agents. The azoles, polyenes, and echinocandins are three main classes of antifungal agents. Amphotericin B (AmB), a polyene discovered in the 1950s, is one of the most effective antifungal agents widely used in clinical practice [2]. However, the severe side effects of AmB, such as fever, nausea, vomiting, rigors, and nephrotoxicity, in some cases results in the premature termination of the treatment [3]. In this respects, it is promising to develop compounds that show synergism with AmB in the treatment of fungal infection diseases. Nicotinamide (NAM), an amide form of vitamin B_3_, is a key NAD+ precursor with long clinical applications due to its safety even at the high dosage. For example, NAM is widely used against pellagra. In a phase III clinical trials, NAM was shown to be useful in preventing skin cancer [4]. Recently, NAM has been reported to show activities against HIV, *Mycobacterium tuberculosis*, *Trypanosoma cruzi* and *Plasmodium falciparum* [5–9]. Our previous research revealed that NAM exhibited significant antifungal activity [10].

In this study, the effect of the combined treatment of NAM and AmB against *C. albicans* was investigated and the underlying mechanisms were explored. We demonstrated the synergistic antifungal interaction of NAM and AmB both *in vitro* and *in vivo*. Moreover, we found that AmB might function through modification of histone acetylation. Thus, addition of NAM, an H3K56 deacetylation inhibitor, could enhance the antifungal activity of AmB.

## Materials and methods

### Strains, medium, and chemicals

The *C. albicans* standard strain SC5314, 21 fungal clinical isolates including *Candida* spp. and *Cryptococcus neoformans* (obtained from Shanghai Changhai Hospital and Skin Disease Hospital, China), *C. albicans* mutant *hst3Δ*/pTET-HST3 and its parent strain CASS1 were used in this study [11]. The strains were cultured in YPD or RPMI 1640 (Invitrogen) medium. The drugs used in this study, including amphotericin B (AmB), nicotinamide (NAM), nystatin and doxycycline (doxy), were all bought from Sigma-Aldrich (St. Louis, MO, USA).

### Antifungal susceptibility assays

The drug susceptibility assay for the fungal cells was performed according to the broth microdilution protocol of the Clinical and Laboratory Standards Institute M27-A3 method with some modification [12]. Briefly, the drugs were diluted twofold in the concentrations ranged from 0.625 to 160 mM for NAM and 0.008 to 4 μg/ml for AmB. The initial cell concentration was 5 × 10^3^ cells/ml. The MIC80 values for the drugs were defined as 80% growth inhibition in the drug-treated groups as compared to the drug-free group. Interpretation of drug interactions were determined on the basis of the fractional inhibitory concentration index (FICI). The value of FICI was expressed as follows: FICI = FICNAM + FICAmB = (MIC80 of NAM in combination/MIC80 of NAM alone) + (MIC80 of AmB in combination/MIC80 of AmB alone). Synergism and antagonism were defined by the FICI of ≤0.5 and >4, respectively. The interaction was defined as indifferent if the FICI was between 0.5 and 4.0.

### Growth curve assay

Growth curve assay was conducted following the procedure described elsewhere [12]. Briefly, the exponentially growing *C. albicans* SC5314 cells were washed and adjusted to 1 × 10^3^ cells/ml in RPMI 1640. Then, the cells were treated with different drugs and cultured at 30°Cwith vigorous shaking. For colony count assay, parts of the cell culture were collected and incubated on YPD agar for two days.

### Determination of antibiofilm activity

The biofilms were formed according to the reference [13]. *C. albicans* SC5314 cells (5 × 10^5^ cells/ml) were allowed to adhere to the substrate (96-well plates) for 90 minutes at 37°C. Then, non-adherent cells were discarded and the fresh medium containing the drugs was added. The plates were further incubated for 24 h at 37°C to allow biofilm formation. XTT assay was used to evaluate biofilm growth. Briefly, biofilm cells were washed with PBS for three times, then 0.5 mg/ml of XTT [2,3-bis (2-methoxy-4-nitro-5-sulfophenyl) −2 H- tetrazolium-5-carboxanilide inner salt] and 1 mM of menadione were added. After incubating at 37°C for 90 min, the optical density (OD) at 490 nm was determined.

### Measurement of biofilm biomass dry weight

The dry weight of biofilm biomass was measured according to the reference with a few modifications [14]. The biofilms were formed on a 12-well culture plate with one silicone disk in each well. After 24 h of biofilm growth, the silicone disks in the wells were collected. Then, the disks were dried at 25°C until the weight did not change any longer. By deducting the original weight of the silicone from the weight for biofilm-formed silicone, the values of the dry weight of biofilm mass were obtained, which were further adjusted for the weight of the silicone squares without cells.

### Determination of the antifungal activity in vivo

To evaluate the *in vivo* antifungal activities of NAM and AmB, a systemic *Candida* infection test was performed. The female BALB/c mice of six weeks old (purchased from the company of Sino-British SIPPR/BK Lab Animal, Shanghai, China) were used in this animal experiment, which was approved by the Animal Ethics Committee of the Second Military Medical University (Shanghai, China). The mice were injected intravenously with 5 × 10^6^ cells/ml of *C. albicans* SC5314 in 200 μl of saline (day 0). Four groups were set up: 3.28 mmol/kg NAM-treated, 0.3 mg/kg AmB-treated, the combined treatment of 3.28 mmol/kg NAM and 0.3 mg/kg AmB and saline-treated (control). At days 0, 1, 2, 4, and 6, the drugs were administered intraperitoneally. For determining the effect of the drugs on the survival of the infected mouse, the mortality of the mouse was monitored daily for 30 days.

For fungal burden assay, the kidneys were collected aseptically four days after infection. After being weighed and homogenized, the mixture was 10-fold diluted and inoculated on YPD agar plates at 30°C for 2 days. The number of the colonies was calculated and expressed as colony forming units (CFU) per mg of kidney.

### Measurement of reactive oxygen species (ROS) production

The determination of the intracellular ROS level was conducted as described previously [15]. The *C. albicans* SC5314 cells grown overnight in YPD liquid medium for 15 h were adjusted to 1 × 10^7^ cells/ml. Then, the fluorescence dye of 20 μg/ml DCFH-DA (purchased from Molecular Probes) was added to the cell suspension and incubated with shaking at 30°C for 30 min. After washing for three times, 10 mM NAM and/or 0.125 μg/ml AmB were added. The culture of the cells was kept on incubating under the same condition. The fluorescence values of the cells were determined with a fluorescence spectrometer (POLARstar Galaxy; BMG Labtech, Offenburg, Germany) at a series of time points, when the cells were harvested and transferred to the wells of a flatbottom microplate (BMG Microplate, 96 well, Black).

### Real-time PCR

The real-time RT-PCR experiment were performed as described previously [16]. The total RNA was extracted from the *C. albicans* SC5314 cells of 10 mM NAM-treated, 0.125 μg/ml AmB-treated, the combined treatment of 10 mM NAM and 0.125 μg/ml AmB and control (drug-free) groups. The primers for the genes detected are listed in Table S1. The mRNA levels were normalized on the basis of their 18S rRNA levels.

### Immunoblotting

The immunoblotting experiment was conducted according to the procedure described previously [17]. Briefly, the exponentially growing *C. albicans* SC5314 cells were exposed to 10 mM NAM, AmB (0.125, 0.25, 0.5 and 1 μg/ml) or the combination of 10 mM NAM and 0.5 μg/ml AmB and cultured at 30°C with vigorous shaking for 4 h. The culture was harvested and the whole-cell proteins were extracted. After being separated on 15% polyacrylamide SDS gels, the proteins were blotted with rabbit anti-H3 (1:4000; Abcam) or rabbit anti-H3K56ac (1:2000; Millipore). After being probed by the secondary antibodies, the proteins were detected by the chemiluminescence method.

### Statistical criteria

The experimental data were analyzed using the GraphPad Prism 6.0 software (San Diego, CA). When the value of *P* < 0.05 or <0.01, it was considered statistically significant.

## Results

### In vitro synergism of NAM and AmB against C. albicans

**Figure 1.**
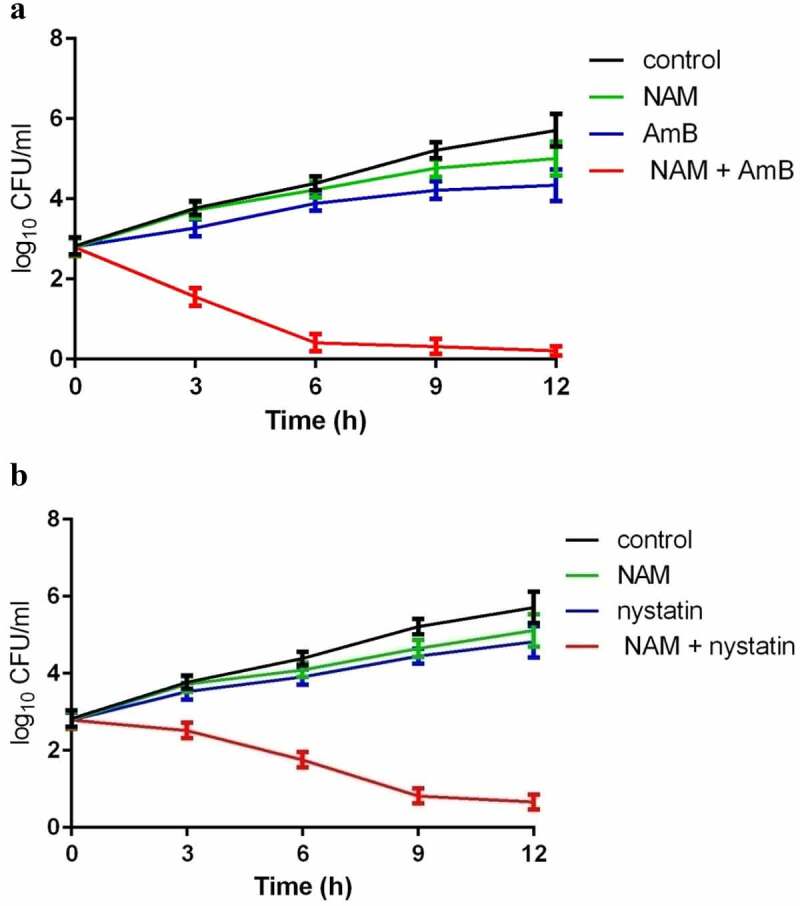
Time-Killing curves of the drugs. (a) the fungal cells were exposed to 5 mM NAM and 0.0313 μg/ml AmB alone or in combination. (b) the fungal cells were exposed to 5 mM NAM and 0.0625 μg/ml nystatin alone or in combination. At the time point of 3, 6, 9 and 12 h, portions of the cell suspensions were harvested and plated on YPD agar to calculate the CFU/ml.

To investigate the interaction of NAM and AmB, the broth microdilution method was performed and the MIC_80_ and FICI were calculated. Either NAM or AmB alone exhibit antifungal activity, while the combined medication of NAM and AmB markedly reduced the values of MIC_80_. The synergism was observed in all of the *C. albicans* clinical isolates listed in the table, with the corresponding FICI ranging from 0.188 to 0.375 (Table 1). The further time-killing test confirmed that NAM could strengthen the antifungal activity of AmB. As shown in Figure 1(a), both 5 mM NAM and 0.0313 μg/ml AmB used alone had slight impact on the growth of the fungal cells, while the cell growth was severely inhibited when 5 mM NAM and 0.0313 μg/ml AmB were added in combination. Combined treatment for 12 h resulted in approximately 4 log10 CFU/ml decrease as compared to the AmB-treated alone group. We next evaluated the effect of NAM on the activity of nystatin, another polyene drug, against *C. albicans*. As shown in Figure 1(b), addition of NAM could remarkably enhance the antifungal activity of nystatin, and the curve representing the combination group showed a remarkable drop as compared to the single drug-treated group.

**Table 1.** Interaction of nicontinamide (NAM) and amphotericin B (AmB) against fungi.

Strains	MIC_80_ alone^a^		MIC_80_ in combination		FIC index
	NAM	AmB	NAM	AmB	
***Candida albicans***					
SC5314	40	0.5	10	0.0625	0.375
691	40	0.25	5	0.0625	0.375
638	80	0.5	10	0.0625	0.25
647	40	0.5	2.5	0.0625	0.313
663	40	0.5	2.5	0.0625	0.313
503	80	0.25	10	0.0313	0.25
531	40	0.25	1	0.0313	0.25
509	80	1	5	0.0625	0.188
562	40	0.5	10	0.0625	0.375
525	40	0.25	5	0.0313	0.25
***Candida tropicalis***					
467	80	0.5	5	0.0625	0.188
411	40	0.5	5	0.0625	0.25
489	80	0.25	10	0.0625	0.375
***Candida glabrata***					
773	40	1	5	0.125	0.25
791	80	1	5	0.125	0.188
741	40	0.5	5	0.0625	0.25
***Candida krusei***					
213	80	1	10	0.5	0.625
204	40	0.5	5	0.125	0.375
266	80	1	10	0.125	0.25
***Cryptococcus neoformans***					
4233	80	2	10	0.125	0.188
4205	80	1	10	0.0625	0.188
4755	80	1	10	0.125	0.25

^a^The unit of nicotinamide (NAM) is mM and the units of amphotericin B (AmB) is μg/ml.

### In vitro synergism of NAM and AmB against a diverse range of fungi

The synergism of NAM and AmB against *C. albicans* prompted us to test the effect of combined treatment of this two drugs on other fungal isolates, including *Candida tropicalis, Candida glabrata, Candida krusei*, and *Cryptococcus neoformans*. As shown in Table 1, similar with the antifungal activity on *C. albicans*, combination of NAM and AmB resulted in a synergistic effect against almost all of the fungal isolates tested, with the highest decrease in MIC_80_ being 16-fold (for NAM, from 80 mM alone to 5 mM in combination; for AmB, from 1 μg/ml alone to 0.0625 μg/ml in combination). These results suggested extensive synergistic antifungal activities for the combination of NAM and AmB.

### NAM enhances the antibiofilm activity of AmB

**Figure 2.**
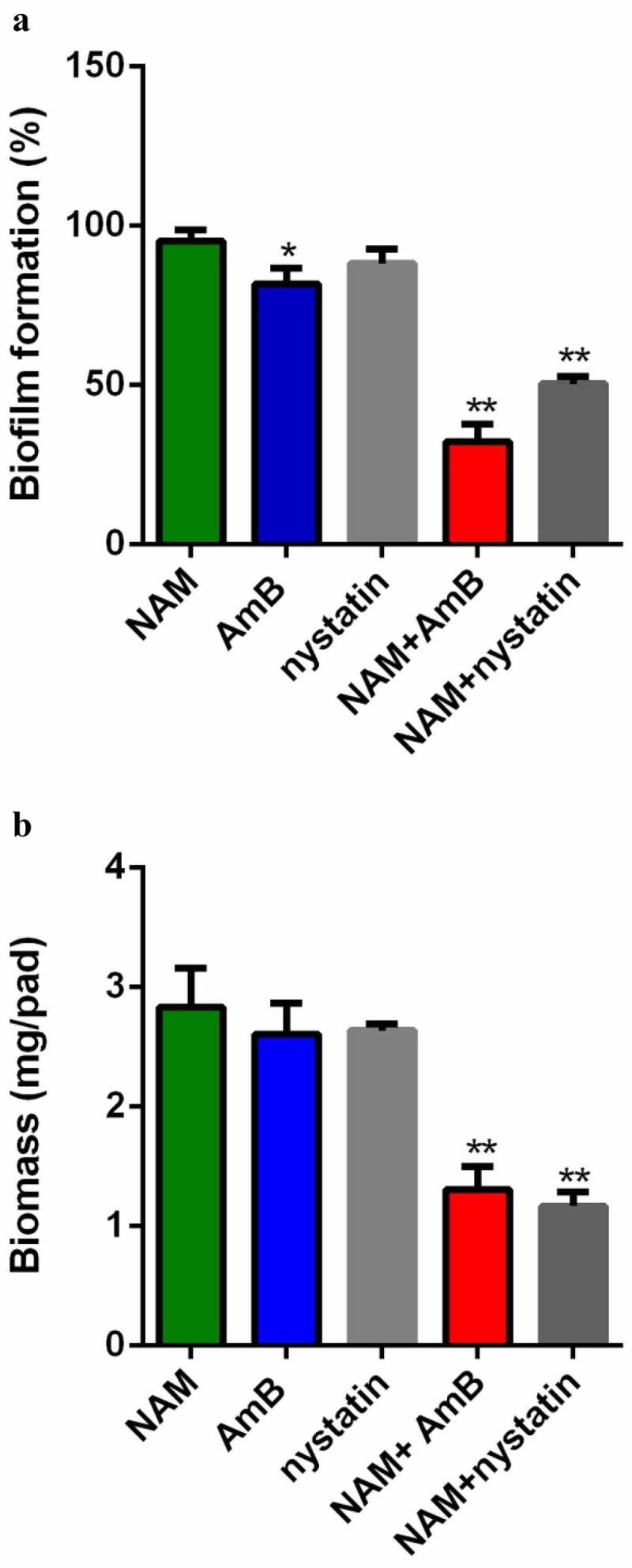
Inhibition of biofilm formation. (a) Effects of 10 mM NAM, 0.5 μg/ml AmB and 0.5 μg/ml nystatin alone or in combination against biofilm. The growth of the biofilm cells were determined by XTT reduction assay. The results are presented as the percent of drug-treated biofilms relative to the control (drug-free) biofilm. (b) Effects of 10 mM NAM, 0.5 μg/ml AmB and 0.5 μg/ml nystatin alone or in combination on biofilms biomass production. *P < 0.05; **P < 0.01 as compared to the control (drug-free) biofilm.

XTT reduction assay was used to evaluate the interaction of NAM and AmB on *C. albicans* biofilm. As shown in Figure 2(a), addition of 10 mM NAM alone did not display a significant antibiofilm activity, while addition of 0.5 μg/ml AmB resulted in a slight inhibitory effect on biofilm formation. However, when 10 mM NAM and 0.5 μg/ml AmB were added in combination, a remarkable inhibition in biofilm formation was observed. Similarly, NAM enhanced the antibiofilm activity of nystatin while 0.5 μg/ml nystatin alone did not significantly inhibit biofilm formation. Consistent with this results, the biofilm biomass test showed that combined treatment of NAM and AmB led to significant decrease in biomass production as compared to the drug treated alone (Figure 2(b)).

### In vivo interaction of NAM and AmB against *C. albicans*

**Figure 3.**
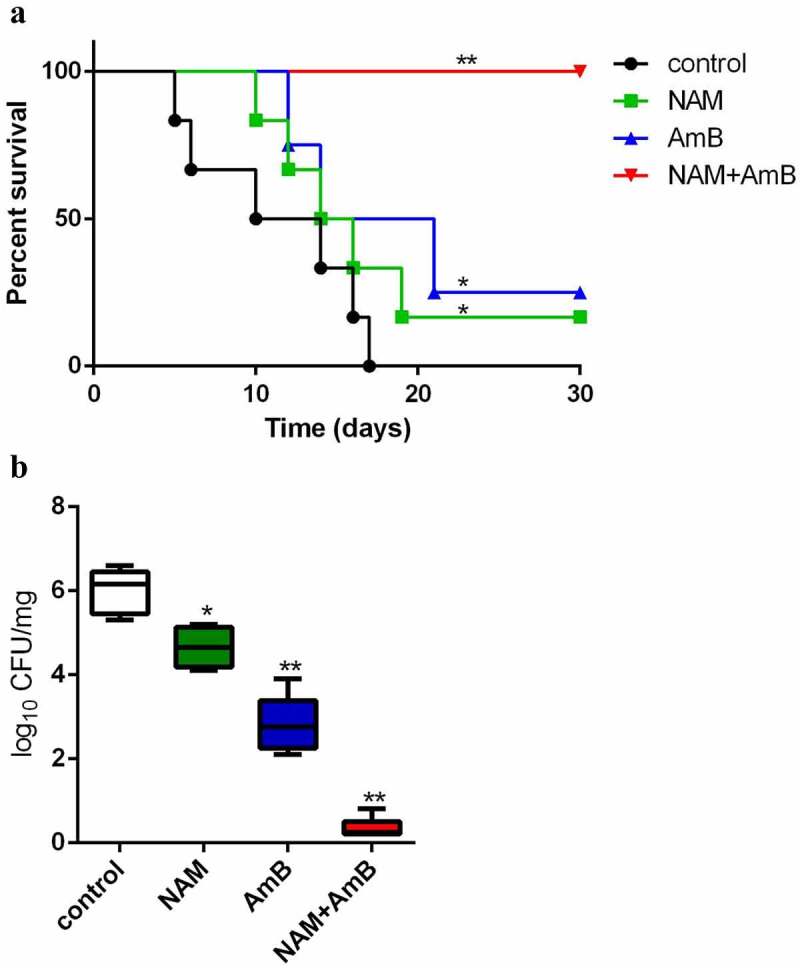
(a) the *in vivo* interaction of NAM and AmB against *C. albicans*. (a) Survival curves of the mice. The BALB/c mice were injected with *C. albicans* SC5314 cells intravenously (0 day). On days 0, 1, 2, 4 and 6, NAM (3.28 mmol/kg) and AmB (0.3 mg/kg) was administered intraperitoneally alone or in combination. The mouse mortality was monitored every day. (b) the mice were sacrificed four days after infection. The kidneys were collected and homogenized. The homogenate was cultured for the calculation of the log reduction in CFU/mg. *P < 0.05; **P < 0.01 as compared to the control (drug-free) group.

Following the synergistic interaction *in vitro*, the *in vivo* interaction of NAM and AmB against *C. albicans* was investigated based on the systemic *Candida* infection test. All of the infected mice in the control group (saline-treated) died within 17 days, while both 3.28 mmol/kg NAM and 0.3 mg/kg AmB alone groups could slightly prolong the survival period of the mice. However, when 3.28 mmol/kg NAM and 0.3 mg/kg AmB were administrated in combination, no mouse was died during the whole observation period, with the survival rate being 100% (Figure 3(a)). Consistently, although both of the single drug-treated groups displayed substantial reduction of fungal loads as compared to the control group, the fungal loads were extremely low when 3.28 mmol/kg NAM and 0.3 mg/kg AmB were administrated in combination (Figure 3(b)).

### Combination of NAM and AMB enhance ROS generation

**Figure 4.**
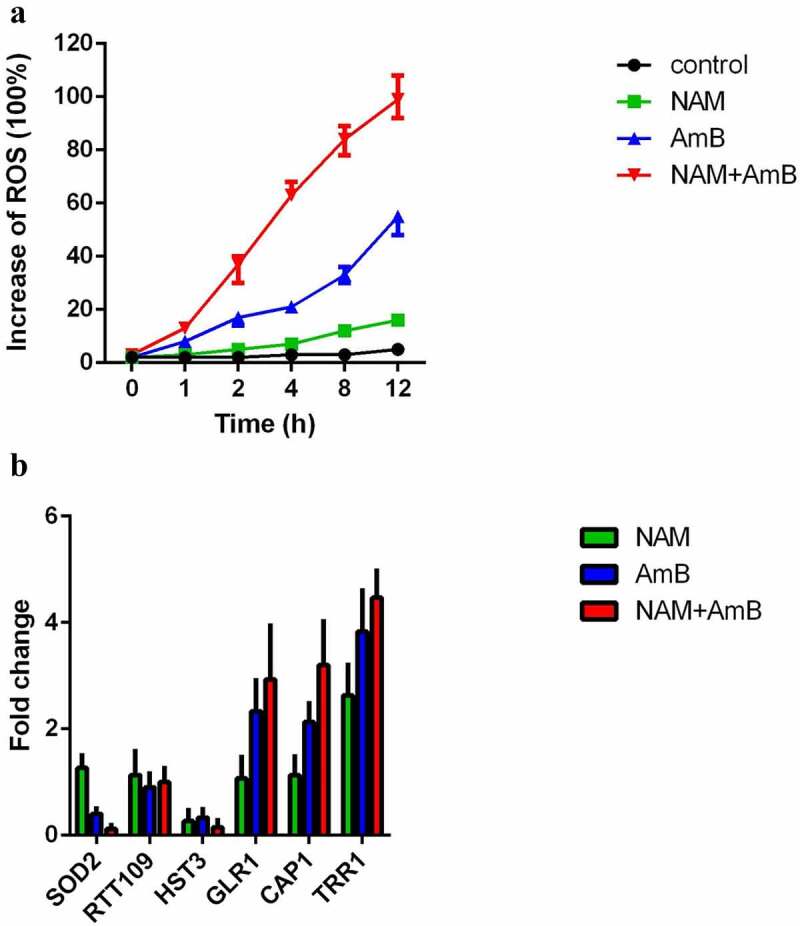
(a) Measurement of ROS production. *C. albicans* SC5314 cells were exposed to 10 mM NAM and 0.125 μg/ml AmB alone or in combination. The ROS level was measured at the indicated time points by a fluorescence spectrometer. The fluorescence value of the combination group at 12 h was considered as 100 % and other fluorescence values were presented as the percent relative to this value. (b) Measurement of mRNA expression. The fungal cells were exposed to 10 mM NAM and 0.125 μg/ml AmB alone or in combination for 4 h. Gene expression was indicated as the fold increase in the drug-treated groups relative to that of the control (drug-free) group.

The induction of intracellular oxidative damage is a fundamental insight in the AMB mode of action. Thus, we evaluated the production of ROS. Addition of 0.125 μg/ml AmB alone could significantly enhance the level of ROS, while 10 mM NAM alone did not significantly affect ROS production. Combined treatment of 10 mM NAM and 0.125 μg/ml AmB led to an even higher intracellular ROS level. At the time point of 12 h, approximately twice higher ROS level was observed in the combination group as compared to the AmB alone group (Figure 4(a)).

We next examined the expression of the genes involved in oxidative stress response. Presence of NAM alone showed up-regulation of TRR1 (a thioredoxin reductase gene). Addition of AmB alone caused up-regulation of TRR1, CAP1 (a transcription factor involved in oxidative stress response), GLR1 (a glutathione reductase gene) and down-regulation of SOD2 (a manganese superoxide dismutase gene). Combination of NAM and AmB caused an even higher expression of TRR1, CAP1, and GLR1 while the expression of SOD2 was severely inhibited (Figure 4(b)).

### AmB inhibits the deacetylation of histone H3 on lysine 56

In *C. albicans*, modification of histone acetylation is considered as an epigenetic control of oxidative stress. NAM is a byproduct during the deacetylation process of histone H3 on lysine 56 (H3K56) and excessive NAM can inhibit this process [11,18]. In view of the enhanced antifungal activity of AmB by NAM, we investigated the effect of AmB on H3K56 acetylation. We first determined the mRNA level of two key enzymes that regulate H3K56 modification, the H3K56 acetyltransferase Rtt109p and deacetylase Hst3p. As shown in Figure 4(b), either NAM or AmB alone or in combination did not affect the expression of RTT109. However, both NAM and AmB alone could down-regulate the expression of HST3, and the expression of HST3 was even lower when NAM and AmB were added in combination.

**Figure 5.**
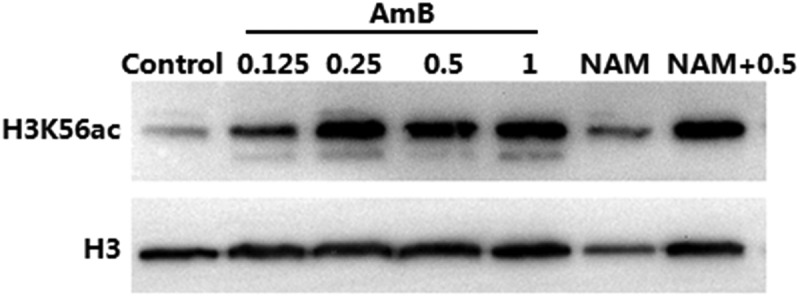
Immunoblot analysis of H3K56ac. *C. albicans* SC5314 cells were treated by 10 mM NAM and AmB (0.125, 0.25,0.5 and 1 μg/ml) alone or the combination of 10 mM NAM and 0.5 μg/ml AmB for 4 h. Then the protein was extracted, separated by SDS gels and probed with rabbit anti-H3K56ac (top) or rabbit anti-H3 (bottom).

We next investigated the level of H3K56ac with immunoblotting. The H3K56ac level in the 0.125 μg/ml AmB-treated group was higher than that in control (drug-free) group. Addition of 0.25 μg/ml AmB resulted in an even higher level of H3K56ac. These results indicated that AmB alone could causes excessive H3K56ac in *C. albicans*. Consistent with the previous report that NAM could inhibit deacetylation of H3K56ac, the H3K56ac level was increased upon exposure to 10 mM NAM alone and the H3K56ac level in the combination group was higher than that in the corresponding drug alone group (Figure 5).

### Inactivation of H3K56 deacetylase Hst3p results in high sensitivity to AmB

**Figure 6.**
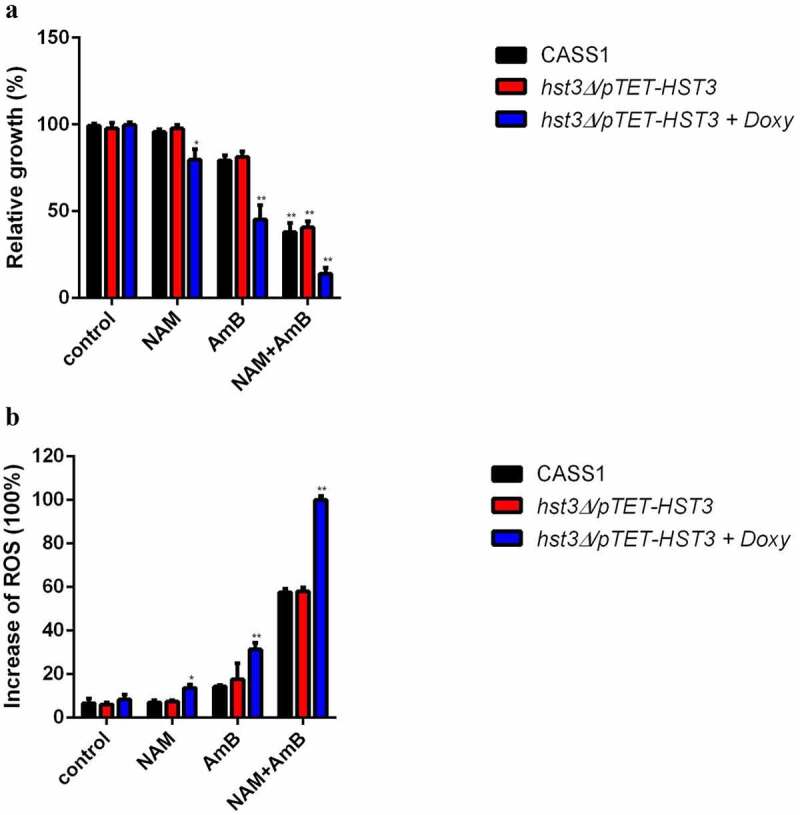
(a) *C. albicans* wild-type strain (CASS1) and *hst3* mutant (hst3δ/ptet-HST3) with or without Doxy were exposed to 10 mM NAM and 0.125 μg/ml AmB alone or in combination for 6 h. The sensitivities of the fungal cells to the drugs were recorded by measuring the OD_600_ values of the cell suspension. The relative growth refers to the ratio of the OD_600_ value of the cells in the drug-treated group to the wild-type strain in the control (drug-free) group. (b) Measurement of ROS production. The fungal cells were exposed to 10 mM NAM and 0.125 μg/ml AmB alone or in combination for 12 h. Then the cell samples were taken for ROS measurement with a fluorescence spectrometer. The fluorescence of the *hst3* mutant with Doxy (hst3δ/ptet-HST3+Doxy) in the combination group was considered as 100 % and other fluorescence values were presented as the percent relative to this value. *P < 0.05; **P < 0.01 as compared to the same strain in the control (drug-free) group.

In view of the down-regulation of HST3 and increased H3K56ac level upon AmB treatment, we further test the role of HST3 upon exposure to AmB. Since HST3 is an essential gene for *C. albicans*, the *hst3Δ*/pTET-HST3 strain, in which the function of HST3 gene is controlled by a promoter that can be inhibited by doxycycline (doxy), was used. As is shown in Figure 6(a), when exposed to AmB alone, the growth of the strain with hst3p inactivation (*hst3Δ*/pTET-HST3 + Doxy) was much slower as compared to the wild type (CASS1) or *hst3Δ*/pTET-HST3 strain without Doxy. Combination of NAM and AmB resulted in an even dramatic decrease in cell growth of the strain with Hst3p inactivation (*hst3Δ*/pTET-HST3 + Doxy). Presence of NAM alone showed a slight inhibition on the growth of the strain with hst3p inactivation.

Since inactivation of Hst3p in *C. albicans* resulted in high drug sensitivity, we next evaluated the intracellular ROS production upon drug treatment. Consistent with the increased drug sensitivity in the Hst3p-inactivated strain (*hst3Δ*/pTET-HST3 + Doxy), the ROS level in this strain was increased upon exposure to NAM, AmB alone or in combination. Of note, both AmB alone or combined with NAM could induce a remarkably increased ROS production in the Hst3p-inactivated strain as compared to the wild type or *hst3Δ*/pTET-HST3 strains without Doxy (Figure 6(b)).

## Discussion

Invasive fungal infections can cause high morbidity and mortality. AmB, which belongs to the polyenes family and was discovered in the 1950s, is a gold standard agent in the therapy of severe invasive fungal infections. However, the well-reported and defined toxicity such as nephrotoxicity hinders the clinical application of this agent. So combination medication is of high importance for AmB because this strategy can enhance efficacy and reduce side effects. Besides, drug repurposing is considered as an immediate and safe therapeutic strategy against fungal infection [19]. Here, we found that NAM, a vitamin with long years of clinical application, could enhance the antifungal efficacy and reduces the dosage of AmB. Our studies revealed that the presence of NAM could strongly enhance the antifungal activity of AmB. The synergistic interaction was observed between NAM and AmB according to the FICI values of these two drugs. It should be noted that the enhanced antifungal activity of AmB by NAM occurred not only in planktonic cells but also in biofilm, which is considered as an important fungal virulence factor due to its high drug resistance. Although the biofilm formed by *C. albicans* is intrinsically resistant to AmB, addition of NAM dramatically enhanced the antibiofilm activity of AmB. Besides, NAM is consistently synergistic with AmB across a wide range of *Candida* spp. as well as *Cryptococcus neoformans*. Moreover, when NAM was used in combination with nystatin, another polyene agent, the remarkably increased activities against planktonic *C. albicans* cells and biofilm were also observed. These results indicated that NAM might be a antifungal sensitizer not restricted to AmB and potentially suitable for multiple antifungal agents.

It has been reported that, in addition to its injurious impacts on fungal membrane, AmB can also induce intracellular ROS production and cause damage to multiple cellular targets such as DNA and proteins [20,21]. A recent study has related the aggregation state of the AmB with the mechanism of action. In aqueous solutions, AmB can be disaggregated and exist mainly as monomers at low concentrations (commonly below the concentration of 1 μg/ml)), or aggregated and exist mainly as dimers and oligomers at high concentrations. The monomeric state of AmB has been proved to be more selective for the ROS production than other aggregation states [22]. In our study, AmB was diluted with the medium to the concentrations ranging from 0.0313 to 1 μg/ml. Thus, the concentrations of AmB used here were low and the aggregation state was mainly monomers, which is consistent with the results obtained in our study. Here increased ROS level was observed in the fungal cells upon exposure to AmB alone, while combination of NAM and AmB caused even more ROS production.

Meanwhile, the expression of several genes related to oxidative stress were changed. In *C. albicans*, Cap1p is a transcription factor responsible for oxidative stress [23]. By exposing the CAP1-depleted *C. albicans* cells to H_2_O_2_, our previous study found seventy-six genes, including several oxidative stress-responsive genes, were differentially expressed [24]. In this study, the expression of CAP1 was induced when the *C. albicans* cells were exposed to AmB alone, and the combination of NAM and AmB caused an even higher expression of CAP1. This can be explained by the cell state. When the ROS is elevated upon endogenous or exogenous stimuli, fungal cells will initiate valid oxidative stress response through up-regulation of the specific transcription factors like Cap1p and thus the overexpression of a series of downstream antioxidant defence-related genes. As expected, GLR1 and TRR1, two genes regulated by CAP1 and encoding the glutathione reductase and thioredoxin reductase, respectively, were up-regulated upon the treatment of AmB alone or in combination. SOD2 encodes a manganese superoxide dismutase, which is a peroxide scavenging enzyme in *C. albicans*. Deletion of SOD2 was found to be hypersensitive to oxidative stress [25]. Here the down-regulation of SOD2 upon the treatment of AmB is suggestive of weakened antioxidant defence in the cells that are consistent with killing of the *C. albicans* cells by AmB.

**Figure 7.**
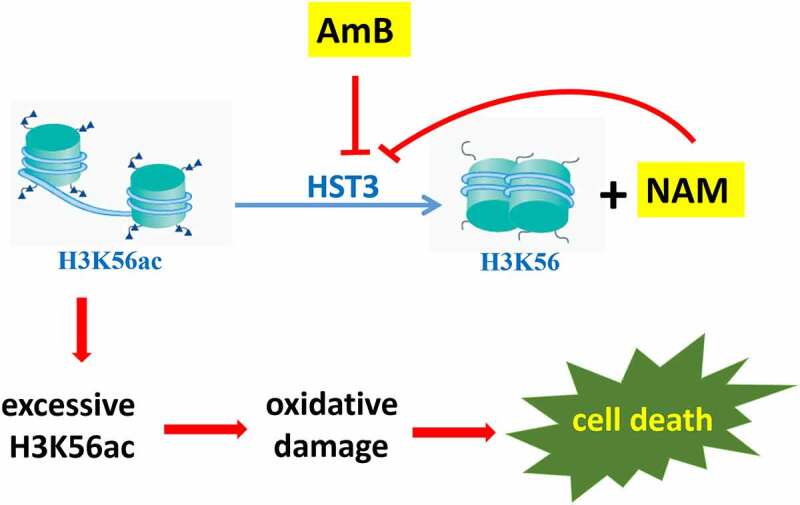
A model explaining the antifungal action of AmB and the synergistic activity between NAM and AmB through epigenetic modification of H3K56ac. AmB could inhibit HST3 expression, which resulted in excessive H3K56ac and the consequent oxidative damage. NAM is a byproduct during the deacetylation course of H3K56ac in *C. albicans*, and excessive NAM can inhibit this process. The combined addition of NAM and AmB led to even excessive H3K56ac and severe oxidative damage, which resulted in increased cell death. Thus enhanced antifungal activity was observed.

It was reported that, in response to ROS, yeast cells operates its defence mechanisms by up-regulation of a series of genes involved in oxidative stress response. Recent studies revealed that, the expression of these genes could be regulated not only by specific transcription factors, but also by epigenetic modifications, including histone acetylation, which were carried out by histone acetyltransferases. Meanwhile, histone deacetylases can remove these acetyl groups [26]. By mmunoprecipitation (ChIP) microarray, Nantel′s group reported that *C. albicans* Ada2p, which was one of the components of the Spt-Ada-Gcn5-acetyltransferase (SAGA) coactivator complex, could associate with the promoters of several genes involved in oxidative stress, and deletion of ADA2 resulted in enhanced sensitivity to oxidative stress [27]. In factor, besides being an amide form of vitamin B_3_, NAM is a byproduct during the deacetylation course of H3K56ac in *C. albicans*, and excessive NAM can inhibit this process. The *C. albicans* H3K56ac is an abundant modification regulated by the acetyltransferase Rtt109p and deacetylase Hst3p [28]. Previous studies revealed that modulation of H3K56ac was required for the virulence and oxidative stress resistance in *C. albicans* [29]. For example, the *rtt109Δ* mutant cannot form normal colonies on the media containing H_2_O_2_, accompanied with increased expression of oxidative stress-response genes. Here we evaluated the expression of RTT109 and HST3 and found that the mRNA levels of RTT109 were not changed upon exposure to the drugs. However, both NAM and AmB alone could severely inhibit the expression of HST3, and this inhibition was even severe when NAM and AmB were added in combination. Moreover, immunoblotting test revealed excessive H3K56ac in AmB-treated groups. These results suggested that HST3 might exert a key role for *C. albicans* against the killing of AmB. As expected, our further experiments showed that inactivation of Hst3p resulted in high sensitivity to AmB, and the even severe growth inhibition was observed when NAM and AmB were added in combination. Consistently, the Hst3p-inactivated strain produced more ROS than the wild type strain upon exposure to the drugs. These findings suggested that AmB might function through epigenetic modification of H3K56ac. That is to say, AmB could down-regulate HST3 expression, which resulted in excessive H3K56ac, while the presence of the H3K56 deacetylation inhibitor NAM could enhance the antifungal activity of AmB (Figure 7).

In conclusion, our studies revealed the synergism between NAM and AmB against *Candida* spp. and *Cryptococcus neoformans*, not only in planktonic cells but also in biofilms. Moreover, the enhanced antifungal activity of AmB by NAM could be observed in a mouse model of disseminated candidiasis. Thus, the combination of NAM and AmB is a promising recipe for antifungal therapy. Our further study revealed a novel mechanism for the antifungal action of AmB, in which epigenetic modification was involved. AmB could execute antifungal activity via promoting Hst3p-mediated H3K56ac. Thus, addition of NAM, an inhibitor of H3K56 deacetylation, led to even excessive H3K56ac and enhanced antifungal activities. This finding provides new ideas for clinical antifungal therapies.

## Data Availability

The authors confirm that the data supporting the findings of this study are available within the article [and/or] its supplementary materials.

## References

[1] Sardi JC, Scorzoni L, Bernardi T, et al. *Candida* species: current epidemiology, pathogenicity, biofilm formation, natural antifungal products and new therapeutic options. J Med Microbiol. 2013;62(1):10–24. DOI:10.1099/jmm.0.045054-0. 23180477

[2] Oura M, Sternberg TH, Wright ET. A new antifungal antibiotic, amphotericin B. Antibiot Annu. 1955;3:566–573. 13355328

[3] Laniado-Laborín R, Cabrales-Vargas MN. Amphotericin B: side effects and toxicity. Rev Iberoam de Micología. 2009;26(4):223–227. 10.1016/j.riam.2009.06.003 19836985

[4] Chen AC, Martin AJ, Choy B, et al. A phase 3 randomized trial of nicotinamide for skin cancer chemoprevention. N Engl J Med. 2015;373(17):1618–1626. DOI:10.1056/NEJMoa1506197. 26488693

[5] Murray MF. Nicotinamide: an oral antimicrobial agent with activity against both *Mycobacterium tuberculosis* and human immunodeficiency virus. Clin Infect Dis. 2003;36(4):453–460. 12567303 10.1086/367544

[6] Sereno D, Alegre AM, Silvestre R, et al. In vitro antileishmanial activity of nicotinamide. Antimicrob Agents Chemother. 2005;49(2):808–812. DOI:10.1128/AAC.49.2.808-812.2005. 15673775 PMC547366

[7] Gazanion E, Vergnes B, Seveno M, et al. In vitro activity of nicotinamide/antil- eishmanial drug combinations. Parasitol Int. 2011;60(1):19–24. DOI:10.1016/j.parint.2010.09.005. 20884376

[8] Prusty D, Mehra P, Srivastava S, et al. Nicotinamide inhibits *Plasmodium falciparum* Sir2 activity in vitro and parasite growth. FEMS Microbiol Lett. 2008;282(2):266–272. DOI:10.1111/j.1574-6968.2008.01135.x. 18397290

[9] Soares MB, Silva CV, Bastos TM, et al. Anti-*Trypanosoma cruzi* activity of nicotinamide. Acta Trop. 2012;122(2):224–229. DOI:10.1016/j.actatropica.2012.01.001. 22281243

[10] Xing XR, Liao ZB, Tan F, et al. Effect of nicotinamide against *Candida albicans*. Front Microbiol. 2019;10:595. 30972047 10.3389/fmicb.2019.00595 PMC6443637

[11] Wurtele H, Tsao S, Lepine G, et al. Modulation of histone H3 lysine 56 acetylation as an antifungal therapeutic strategy. Nat Med. 2010;16(7):774–780. DOI:10.1038/nm.2175. 20601951 PMC4108442

[12] Quan H, Cao YY, Xu Z, et al. Potent in vitro synergism of fluconazole and berberine chloride against clinical isolates of *Candida albicans* resistant to fluconazole. Antimicrob Agents Chemother. 2006;50(3):1096–1099. DOI:10.1128/AAC.50.3.1096-1099.2006. 16495278 PMC1426442

[13] Ramage G, Vande Walle K, Wickes BL, et al. Standardized method for in vitro antifungal susceptibility testing of *Candida albicans* biofilms. Antimicrob Agents Chemother. 2001;45(9):2475–2479. DOI:10.1128/AAC.45.9.2475-2479.2001. 11502517 PMC90680

[14] Nobile CJ, Andes DR, Nett JE, et al. Critical role of Bcr1-dependent adhesins in *C. albicans* biofilm formation in vitro and in vivo. PLoS Pathog. 2006;2(7):e63. DOI:10.1371/journal.ppat.0020063. 16839200 PMC1487173

[15] Li DD, Chai D, Huang XW, et al. Potent in vitro synergism of fluconazole and osthole against fluconazole-resistant *Candida albicans*. Antimicrob Agents Chemother. 2017;61(8):e00436–17. DOI:10.1128/AAC.00436-17. 28607012 PMC5527582

[16] Yan Y, Tan F, Miao H, et al. Effect of shikonin against *Candida albicans* biofilms. Front Microbiol. 2019;10:1085. 31156594 10.3389/fmicb.2019.01085 PMC6527961

[17] Liao ZB, Zhu ZY, Li L, et al. Metabonomics on *Candida albicans* indicate the excessive H3K56ac is involved in the antifungal activity of Shikonin. Emerg Microbes Infect. 2019;8(1):1243–1253. DOI:10.1080/22221751.2019.1657362. 31452461 PMC6735332

[18] Celic I, Masumoto H, Griffith WP, et al. The sirtuins hst3 and Hst4p preserve genome integrity by controlling histone h3 lysine 56 deacetylation. Curr Biol. 2006;16(13):1280. DOI:10.1016/j.cub.2006.06.023. 16815704

[19] Mei Y, Jiang T, Zou Y, et al. FDA approved drug library screening identifies robenidine as a repositionable antifungal. Front Microbiol. 2020;11:996. 32582050 10.3389/fmicb.2020.00996 PMC7283467

[20] Sokol-Anderson MJE, Sligh JS, Elberg J, et al. Role of cell defense against oxidative damage in the resistance of *Candida albicans* to the killing effect of amphotericin B. Antimicrob Agents Chemother. 1988;32(5):702–705. DOI:10.1128/AAC.32.5.702. 3293525 PMC172255

[21] Sokol-Anderson ML, Brajtburg J, Medoff G. Amphotericin B-induced oxidative damage and killing of *C. albicans*. J Infect Dis. 1986;154(1):76–83. 3519792 10.1093/infdis/154.1.76

[22] Fernández-García R, Muñoz-García JC, Wallace M, et al. Self-Assembling, supramolecular chemistry and pharmacology of amphotericin B: poly-aggregates, oligomers and monomers. J Control Release. 2022;341:716–732. 34933052 10.1016/j.jconrel.2021.12.019

[23] Moye-Rowley W. Regulation of the transcriptional response to oxidative stress in fungi: similarities and differences. Eukaryot Cell. 2003;2(3):381–389. 12796283 10.1128/EC.2.3.381-389.2003 PMC161443

[24] Wang Y, Cao Y, Jia X, et al. Cap1p is involved in multiple pathways of oxidative stress response in *Candida albicans*. Free Rad Biol Med. 2006;40(7):1201–1209. DOI:10.1016/j.freeradbiomed.2005.11.019. 16545688

[25] Hwang CS, Baek YU, Yim HS, et al. Protective roles of mitochondrial manganese-containing superoxide dismutase against various stresses in*Candida albicans*. Yeast. 2003;20(11):929–941. DOI:10.1002/yea.1004. 12898709

[26] Marmorstein R, Zhou M. Writers and readers of histone acetylation: structure, mechanism, and inhibition. Cold Spring Harb Perspect Biol. 2014;6(7):a018762. 24984779 10.1101/cshperspect.a018762 PMC4067988

[27] Sellam A, Askew C, Epp E, et al. Genome-Wide mapping of the coactivator Ada2p yields insight into the functional roles of SAGA/ADA complex in *Candida albicans*. Mol Biol Cell. 2009;20(9):2389–2400. DOI:10.1091/mbc.e08-11-1093. 19279142 PMC2675619

[28] Dahlin JL, Chen X, Walters MA, et al. Histone modifying enzymes, histone modifications and histone chaperones in nucleosome assembly: lessons learned from Rtt109 histone acetyltransferases. Crit Rev Biochem Mol Biol. 2015;50:31–53. 25365782 10.3109/10409238.2014.978975 PMC4415165

[29] da Rosa L, Boyartchuk VL, Zhu LJ, et al. Histone acetyltransferase Rtt109 is required for *Candida albicans* pathogenesis. Proc Natl Acad Sci USA. 2010;107(4):1594–1599. DOI:10.1073/pnas.0912427107. 20080646 PMC2824404

